# Supplementary material for: Health-Related Quality of Life in Locally Advanced Gastric Cancer: A Systematic Review
Source: Cancers (Basel). 2021 Nov 25;13(23):5934. doi: 10.3390/cancers13235934 (PMC8657098; doi:10.3390/cancers13235934)
Supplement: Supplementary file 1 [file cancers-13-05934-s001.zip › cancers-1448967-supplementary.pdf]

cancers MDPI

Supplementary data

Search strategy

Table S1. Embase search:

No.	Query	Results
#5	#1 AND #2 NOT (#4 NOT #3)	4394
#4	'palliative therapy'/exp OR 'palliative therapy' OR palliat* OR 'hospice'/exp OR hospice OR (((symptomatic OR supportive) NEAR/3 (treatment* OR therap* OR care)):ti,ab,kw)	284874
#3	curat*:ti,ab,kw OR 'non metast*':ti,ab,kw OR nonmetast*:ti,ab,kw OR 'local advance*':ti,ab,kw OR 'locally advance*':ti,ab,kw OR current*:ti,ab,kw OR 'long term':ti,ab,kw OR longterm:ti,ab,kw OR 'stage 1':ti,ab,kw OR 'stage 2a':ti,ab,kw OR 'stage iia':ti,ab,kw OR 'life prolong*':ti,ab,kw OR 'life sustain*':ti,ab,kw	3578324
#2	'patient-reported outcome'/exp OR 'patient reported outcome measure'/exp OR 'patient reported outcome measurement'/exp OR 'patient reported outcome questionnaire'/exp OR 'quality of life'/exp OR 'quality of life assessment'/exp OR ('patient reported':ti,ab,kw AND outcome*:ti,ab,kw) OR 'patient outcome assessment*':ti,ab,kw OR 'patient centered outcome*':ti,ab,kw OR 'patient centred outcome*':ti,ab,kw OR prom:ti,ab,kw OR proms:ti,ab,kw OR 'quality of life':ti,ab,kw OR hrqol:ti,ab,kw OR qol:ti,ab,kw OR (('self report*' NEAR/6 outcome*):ti,ab,kw) OR (((symptom* OR functioning OR 'self report*') NEAR/6 (surveillance OR supervision* OR monitor*)):ti,ab,kw)	713150
#1	'stomach cancer'/exp OR 'stomach cancer' OR (((gastric OR stomach) NEAR/6 (neoplas* OR malignan* OR tumor* OR tumour* OR cancer* OR carcino* OR adenocarcino*)):ti,ab,kw)	183098
**Medline search:**		
1	exp Patient Reported Outcome Measures/ or Patient Outcome Assessment/ or Outcome Assessment, Health Care/ or Quality of Life/ or (("patient reported" and outcome*) or "patient outcome assessment*" or "patient centered outcome*" or "patient centred outcome*" or prom or proms or "quality of life" or hrqol or qol or ("self report*" adj6 outcome*) or ((symptom* or functioning or self-report*) adj6 (questionnaire* or construct* or interview* or scale* or instrument* or tool* or surveillance or supervision* or monitor*))).ti,ab,kf.	540591
2	exp Stomach Neoplasms/ or ((gastric or stomach) adj6 (neoplas* or malignan* or tumor* or tumour* or cancer* or carcino* or adenocarcino*)).ti,ab,kf.	137099
3	(curat* or "non metast*" or nonmetast* or "local advance*" or "locally advance*" or current or "long term" or longterm or "stage 1" or "stage 2a" or "stage iia" or "life prolong*" or "life sustain*").ti,ab,kf.	2251183
4	exp Palliative Care/ or exp Hospices/ or (palliat* or hospice or ((symptomatic or supportive) adj3 (treatment* or therap* or care))).ti,ab,kf.	157181
5	(1 and 2) not (4 not 3)	2463

Scopus search:

((TITLE-ABS (((gastric OR stomach) W/6 (neoplas* OR malignan* OR tumor* OR tumour* OR cancer* OR carcino* OR adenocarcino*))) OR AUTHKEY (((gastric OR stomach) W/6 (neoplas* OR malignan* OR tumor* OR tumour* OR cancer* OR carcino* OR adenocarcino*)))) AND (TITLE-ABS ((("patient reported" AND outcome*) OR "patient outcome assessment*" OR "patient centered outcome*" OR "patient centred outcome*" OR prom OR proms OR "quality of life" OR hrqol OR qol OR ("self report*" W/6 outcome*) OR ((symptom* OR functioning OR self-report*) W/6 (questionnaire* OR construct* OR interview* OR scale* OR instrument* OR tool* OR surveillance OR supervision* OR monitor*)))) OR AUTHKEY ((("patient reported" AND outcome*) OR "patient outcome assessment*" OR "patient centered outcome*" OR "patient centred outcome*" OR prom OR proms OR "quality of life" OR hrqol OR qol OR ("self report*" W/6 outcome*) OR ((symptom* OR functioning OR self-report*) W/6 (questionnaire* OR construct* OR interview* OR scale* OR instrument* OR tool* OR surveillance OR supervision* OR monitor*)))))) AND NOT ((TITLE-ABS(palliat* OR hospice OR ((symptomatic OR supportive) W/3 (treatment* OR therap* OR care))) OR AUTHKEY((palliat* OR hospice OR ((symptomatic OR supportive) W/3 (treatment* OR therap* OR care))))) AND NOT (TITLE-ABS(curat* OR "non metast*" OR nonmetast* OR "local advance*" OR "locally advance*" OR current OR "long term" OR longterm OR "Stage 1" OR "stage 2a" OR "stage IIa" OR "life prolong*" OR "life sustain*") OR AUTHKEY((curat* OR "non metast*" OR nonmetast* OR "local advance*" OR "locally advance*" OR current OR "long term" OR longterm OR "Stage 1" OR "stage 2a" OR "stage IIa" OR "life prolong*" OR "life sustain*"))))
